# Bringing the autistic lifeworld to supportive technology design: an enactive approach

**DOI:** 10.1080/15710882.2023.2295952

**Published:** 2023-12-28

**Authors:** Johannes Cornelis van Huizen, Jelle van Dijk, Wouter G. Staal, Mascha C. van der Voort

**Affiliations:** a Human-Centred Design, University of Twente, Enschede, Netherlands; b Faculty of Social Sciences, University of Leiden, Leiden, Netherlands

**Keywords:** Autism, supportive technology, enactivism, lifeworld

## Abstract

Supportive technologies for autistic individuals are promising in principle, yet their uptake remains limited. Critics argue that in current designs of supportive technologies, autism is mostly framed as a ‘disorder’ whose limitations can be pragmatically compensated for. To increase uptake, designers should get a better handle on how to incorporate the full richness of the autistic experience into the design process. This paper presents an integrative framework of the autistic lifeworld, called *Autistic Lifeworld Design* (hereafter: ALD). ALD evolved in a transdisciplinary research setting, substantiated by 11 design case studies with autistic young adults as well as theoretical inquiries into enactivism, design and autism. It consists of four dimensions of experience – *sensory*, *habitual*, *social*, and *affective* –, each providing specific pointers on how to better understand how autistic people experience the world and how supportive technologies may complement that experience. By adopting an enactive approach, ALD enables a reframing of supportive technology as helping to sustain different levels of homoeostasis. It offers a novel lens that allows designers to put the lived experiences of autistic individuals at the centre of the design process, with special attention to the role of bodily structures and processing in shaping these experiences.

## 1. Introduction

Approximately 1% of the global population is estimated to be autistic (Zeidan et al. 2022), a condition medically defined by impairment in stimulus processing, executive functioning and social interaction (American Psychiatric Association 2013; Marco et al. 2011). Whether autism is understood as a disorder or manifests as a disability depends on both context and culture.^1^ Regardless, being autistic typically leads to additional challenges in life, including poor outcomes in education and professional work and a higher prevalence of depression and burnout (Kapp, Gantman, and Laugeson 2011).

One way to meet some of these challenges is through *supportive technology*. Supportive technology refers to ‘any device or piece of equipment that facilitates teaching new skills, augments existing skills, or otherwise reduces the impact of disability on daily functioning’ (Lang et al. 2014, 157). Supportive technology may be ‘low-tech’, e.g. visual schedules (Ganz 2007), or ‘high-tech’ (Shane et al. 2012), e.g. VR-environments supporting emotion recognition and regulation (Mesa-Gresa et al. 2018). The use of supportive technologies promises a two-fold advantage over traditional services: they reduce healthcare costs and allow users to exert more control over aspects of their own life.

### 1.1. Supportive technology design and the deficit model of autism

Supportive technologies meant to support autistic individuals are promising in principle, yet their uptake remains limited (Fletcher-Watson 2014; Zervogianni et al. 2020). A growing critique is that, in the design of supportive technologies, the focus is too strongly on limitations assumed in the deficit model of autism (Frauenberger 2015; Rapp et al. 2018). For instance, Desideri et al. (2020) discuss technological interventions specifically aimed at compensating for deficits related to executive functions, e.g. mobile applications supporting planning and task management. In addition, many tools are designed to train or teach autistic people – children, typically – skills that they supposedly lack. For example, social robot ‘Probo’ is designed to improve verbal utterances and eye contact among autistic children (Pop et al. 2014). And Syriopoulou-Delli and Gkiolnta (2019) report on numerous robots aimed at reducing repetitive and stereotypical behaviours. Frauenberger, Good and Pares write:

[Supportive technologies] are fundamentally driven by a deficit model of disability, and focus on the mitigation of a functional limitation. However, such a model ignores the rich and complex life-worlds of people with autism as an opportunity space for design. (2016, 3347)

In recent years, advocates of the neurodiversity movement have argued for a different perspective on autism, characterising it as a biological *difference* rather than a disorder (Kapp et al. 2013; Singer 1999). This provides a much-needed corrective to the deficit model of autism, and enables us to rethink how to design supportive technology by putting the lived experiences of autistic people at the centre of to the design process.

### 1.2. Bringing the autistic lifeworld to supportive technology design

A crucial concept in this context is that of the lifeworld, which evolved in phenomenology (Agamben 2004; Heidegger [1927] 2010; Husserl (1936) 1970; Merleau-Ponty [1945] 2013; Schutz and Luckmann 1973) and slowly made its way into interaction design (Dourish 2001). The lifeworld is used to distinguish between the world as described objectively from an external point of view, and the world as is it subjectively experienced by each individual. In this regard, the lifeworld focuses not so much on autism’s diagnostic features but on how the world shows up for an autistic person as meaningful. Likewise, the lifeworld prompts designers to create supportive technologies not so much to compensate for deficits, but at doing justice to the full experience of being autistic.

The *OutsideTheBox-*project by Frauenberger, Spiel, and Makhaeva (2019, 2017) and the *MyDayLight*-project by Van Dijk et al. (2016, 2017) are two noteworthy examples of how the lifeworld is used in design practice, stimulating the use of participatory methods that focus on context and interaction. Frauenberger, Spiel and Makhaeva (2019, 666) aimed to ‘holistically respond to the complex lifeworlds of autistic children’, using generative workshops and theatre methods, among others, to create meaningful technologies that match their participants’ interests – e.g. a reflection device inspired by science and technology and a wake-up system inspired by SuperMario, respectively. Van Dijk et al. provided participants with a light system consisting of one or multiple units to function as cues for action; to augment existing meaningful structure in the lifeworld rather than replace it with externally normative ‘schedules’, ‘plan boards’ and ‘task planners’. It was observed that no participant used the light system in the same way, but rather in sync with or in relation to idiosyncratic routines – e.g. a reminder to do laundry or rest, ‘hooking on’ the user’s existing lifeworld (2019, 13).

Building on the work of Frauenberger, Spiel, Makhaeva and Fitzpatrick (2019, 2017) and the work of Van Dijk et al. (2016, 2017, 2019), the main aim of this paper is to provide an integrated framework that enables designers and autistic individuals to analyse and reflect on the latter’s lived experiences, and bring them into the design space. We argue, however, that the framework should also accommodate and reconcile with insights from autism-specific scientific research, such as on hypo- and hypersensitivity, patterns of behaviour, and the importance of special interests, among others (Boldsen 2018, 907). Autism research has historically been driven by the deficit model of autism (Dyck and Russell 2020; Kapp et al. 2013), which means that we need to revalue autism research so that it can help designers to truly meet the needs and concerns of autistic individuals.

One way to focus on the lived experiences of autistic individuals while also integrating autism-specific scientific research is to adopt an *enactive approach*. Enactive philosophy foregrounds the lived experiences of individuals, but with a particular focus on the role that bodily processes play in shaping these experiences (Varela, Thompson, and Rosch 1991). De Jaegher (2013, 6) positions enactivism as a ‘dialogue between phenomenology and science’. Applied to supportive technology design, an enactive approach can thus contribute to a design framework grounded in the situated and embodied nature of autistic experiences while also taking into account insights from autism research.

### 1.3. Paper purpose and structure

The integrative framework proposed in this paper is called *Autistic Lifeworld Design* (ALD). Using enactivism as a foundation, the question it seeks to answer is: how exactly should designers understand the autistic lifeworld, and how can they account for it in a participatory design process? As will be elaborated below, the ALD-framework that resulted from our research contains four dimensions: *sensory*, *habitual*, *social* and *affective*, each providing pointers on how autistic people experience the world and how supportive technologies can meaningfully complement that experience.

This paper is structured as follows. First, Section 2 briefly introduces the lifeworld itself, drawing in particular on the work of De Jaegher (2013). After that, Section 3 details the transdisciplinary approach to the content of this paper. ALD has been informed both by theoretical inquiries into enactivism, design and autism research, as well as by the lived experiences of autistic young adults gathered from 11 design case studies. Section 4 presents the ALD-framework, and Section 5 provides methodological reflections and directions for future research.

## 2. The lifeworld and embodied sense-making

According to enactivism, experience should be understood as a form of *sense-making* (De Haan 2020; Varela, Thompson, and Rosch 1991). Sense-making applies to any human being and refers to the process of interacting with the world while discovering which of its elements are meaningful to you and which ones are not. Consider, for instance, a blind and a deaf person waiting at a crosswalk. For the blind person, it is the acoustic and tactile signal generators of the traffic lights that are meaningful to them; for the deaf person, it is the visual signal generator that matters to them. The blind and the deaf person both cast a ‘web of significance’ on the same environment, yet whatever catches their attention will not be the same (De Jaegher 2013, 6). Although they inhabit the same physical space, they adapt to it in different ways.

### 2.1. When is something ‘meaningful’?

Enactivism grounds the process of sense-making in the process of life itself – that is, in the process of sustaining one’s sovereignty over and against events in the environment. Therefore, the experienced meaningfulness of elements therein can ultimately be traced back to the most basic ways in which they are either advantageous to one’s *autonomy* or, on the contrary, *threaten* it (Maturana and Varela 1980). This is what makes it possible for elements in the world to ‘matter’ to us. Referring to sense-making as an organism’s ‘evaluative interaction with its environment’, De Haan describes it as follows:

In sense-making, there is something at stake. The fundamental dependency of organisms on their surroundings implies that the interaction between organism and world is not neutral: the organism has specific needs and concerns and what the environment offers is evaluated accordingly. (2020, 56)

The evaluation that De Haan refers to takes place on multiple levels, and mostly happens unconsciously. At the biological level, a person constantly exchanges ‘matter and energy with the environment’ and yet the body is able to recognise which ones of these interactions are beneficial and which ones are not (De Jaegher 2013, 5).

The notion of autonomy also applies to the social domain, although the term ‘needful freedom’ better captures the social dynamics it seeks to describe (Jonas 1966; Loaiza 2019, 25). A kind of social homoeostasis can be observed: the self-organising patterns through which people maintain their relative autonomy in interaction with others. Indeed, we do not only continuously interact with ‘matter and energy’, but also with other people – we are *inter*dependent, through work, school, care and other forms of social interaction. And while we recognise the value of this interdependence for our well-being, we care to ensure that a stable balance is maintained between the level of independence that one gives away and the level of freedom that one preserves.

Finally, autonomy also plays out on a more affective level. When interacting with the physical and social environment, we experience all kinds of *feelings*. For example, we ‘feel’ at home when posters and souvenirs are displayed on the wall. They resemble interests and hobbies and evoke a sense of pleasure. Photos of loved ones provoke fond memories from the past, but they can also trigger fears about how the future will unfold. Here, Heersmink (2018, 1846) makes the connection to personal identity, stating that ‘[our] embodied interactions with evocative objects trigger and sometimes constitute emotionally-laden autobiographical memories, which are the building blocks for our narrative’. This has normative implications, Heersmink (2017, 2018, 1846) argues, insofar as external artefacts can support a sense of personhood – ‘if objects constitute who we are, then those objects ought not to be interfered with’ – but can also keep alive an experience one rather forgets. In short, human beings and their surroundings connect on an emotional plane, and here too a stable balance should be sustained – a form of ‘affective homeostasis’, that is.

In essence, then, sense-making signifies a form of evaluative interaction with the world that is geared towards the satisfaction of needs and the mitigation of concerns. It means to recognise and withstand elements within your environment that could harm you at different levels of homoeostasis. As a living system, De Jaegher (2013, 5) concludes, the body is ‘a precarious network of various interrelated self-sustaining identities (organic, cognitive, social), each interacting with the world in terms of the consequences of its own viability’.

### 2.2. How can technologies ‘support’ sense-making?

Enactivism allows us to reframe the way technologies can be ‘supportive’, namely by helping to sustain different levels of homoeostasis. When waiting at a crosswalk, the blind person uses their cane to safely guide themself through the ridged pavement. When at home, the deaf person might use sign language – a technology too, in this context – to communicate with others about their needs, concerns and expectations. In these examples, the cane and sign language have seamlessly and purposefully integrated into their user’s *lifeworld*. They are ‘supportive’ not because they compensate for an externally and normatively defined deficit, but because they *mediate* self-sustaining processes of sense-making that are authentic and meaningful for the sense-maker (De Jaegher 2013; Verbeek 2005).

If a supportive technology fails to integrate into the user’s lifeworld, it is because the technology evokes aversion – or, to stay in enactivist terminology, something in or about its design is experienced as a ‘threat’ to one’s autonomy. Sensorially, habitually, socially or affectively, the supportive technology triggers a need of concern that prevents integration: the cane might be too short, the sign language might be incorrect, etc. In these cases, the cane and sign language do not mediate processes of sense-making because they are themselves *subjects* of sense-making.

In this respect, one explanation for the limited uptake of current supportive technologies is that the lifeworld of a non-autistic designer has limited qualitative overlap with the lifeworld of an autistic end user (Spiel, Frauenberger, and Fitzpatrick 2017). Designers struggle to empathise with end users’ needs and concerns, again at sensory, habitual, social and affective levels. Instead, when making design choices, designers often implicitly rely on their own neurotypical ways of interacting within their own lifeworld, or they turn to an explanation of autistic ways of sense-making according to the deficit model. However, these neurotypical ways of looking at autistic sense-making run the risk of producing technologies that evoke aversion in interaction with end users.

### 2.3. What is the ‘autistic lifeworld’?

In the examples given above, it should be observed that one’s bodily make-up influences the interpretation of what is meaningful and what is not.

With reference to the above, our starting point for a characterisation of the autistic lifeworld is therefore the autistic *embodiment*. As De Jaegher (2013, 8) describes it: ‘the particular ways in which the biology, neurophysiology, affective, and sensorimotor structures and skills of people with autism differ from those of non-autistic individuals’. Similar to blind and deaf people, autistic people too cast a ‘web of significance’ over their environment that is grounded in their particular embodiment. Whatever catches their attention, affords action, and is perceived as useful, threatening, overwhelming, chaotic, clear, is influenced by their bodily make-up. Thus, auditory notifications in mobile applications may be counterproductive as they can cause sensory overstimulation. Visual cues may be meaningful as they can bring structure and evoke calm feelings, or vice versa, can distract and produce unrest. Autistic embodiment leads to a distinct type of sense-making and this results in distinct needs and concerns at sensory, habitual, social and affective levels. Combined, these needs and concerns constitute the *autistic lifeworld*.

On the one hand, autistic embodiment seems to show certain overall patterns that many autistic individuals share, such as, for example, a focus on detail rather than wholes in perception. On the other hand, autistic experiences are extremely heterogeneous, and each autistic person may have very specific ways of responding to and dealing with the world.

With this theoretical background at hand, the question for ALD becomes the following: which of the above needs and concerns are relevant for design, and how can they be accounted for in a participatory design process? This question was addressed in a multi-case study setting with autistic young adults. In what follows, we outline the research setting and methodical procedures.

## 3. Method

### 3.1. Research context, participants and case study protocol

In total, we conducted 11 design case studies with autistic young adults (Table 1), mostly facilitated by Bachelor- and Master industrial design students as part of their final projects. These case studies were conducted in the Netherlands between July 2020 and June 2022, all within the context of *Design Your Life*. This is an ongoing design research initiative developing a co-design toolkit to help autistic young adults create their own supportive technologies (Waardenburg et al. 2022).

**Table 1.** An overview of the case studies, including age, living situation, design partner and duration. Participants are referred to using pseudonyms.

Participant (age)	Living Situation	Design Partner	Duration (months)
Anne (16)	Living with parents.	Professional caregiver and mother.	2
Herman (32)	Living on his own;	Partner.	8
Renée (18)	Partly living at a mental healthcare organisation, partly living at home.	Two professional caregivers.	8
Anton (39, outlier in age)	Living on his own.	Anton did not choose a design researcher and worked independently.	8
Simon (26)	Living at mental healthcare organisation.	Professional caregiver.	7
Sky (17)	Living with parents.	Parents and two professional caregivers.	3
Willem (18)	Living with his parents.	Design researcher.	5
Multiple Participants (25–32)	Living with parents and mental healthcare organisation.	Parents and professional caregiver.	3
Vincent (23)	Living at mental healthcare organisation.	Professional caregiver.	3
Paul (33)	Living at mental healthcare organisation.	Professional caregiver.	3
Tim (14)	Living at home.	Parent.	3

Our participants were aged between 14 and 40, with an IQ of higher than 70.^2^ Nine participants were recruited through mental healthcare organisations within our consortium, while the other participants were approached through personal social networks. This study was approved by the Ethics Committee Natural Sciences and Engineering Sciences of the University of Twente (reference number: *2020.44*). In compliance, participants signed a physical consent form explaining what participation entailed and how the research data would be stored and processed.

Although the case studies differed in research focus and duration, a case study protocol was established in advance to ensure a base level of uniformity. All participants chose a *design partner* to support them in the design process – be it a professional caregiver, relative or partner. In addition, the participants followed the same six-step design procedure, going through phases of context-mapping, ideation, conceptualisation, realisation, prototype deployment and evaluation. Each phase provided the participant with one or more design tool(s) associated with that phase – e.g. a photograph activity in the context-mapping phase to map supportive technologies already in use, a tinkering activity using Velcro and ‘buttons’ in the conceptualisation phase, and an assessment matrix to highlight (dis)satisfaction with the end product in the evaluation phase. The six-step design procedure of Design Your Life has been described in more detail in earlier work (Van Huizen et al. 2022).

### 3.2. Data collection

All case studies were practice-oriented and led to the collection of qualitative data: interviews related to the research question, observations, researcher logbooks and reflections, as well as the resultant technologies themselves and the outcomes of intermediate design activities. In addition, data was discussed and collected during three multi-stakeholder meetings with experience experts as well as caregivers, autism coaches and healthcare managers from the clinical context.

All data is structured and displayed on a physical ‘data wall’, with virtually no data left behind either. High-resolution photographs of the data wall can be accessed here, providing more in-depth information about the participants and their design activities: https://app.mural.co/t/dyl2140/m/dyl2140/1692266984070/7f232d361f07f06d013af17d541402f03e9e0ca4?sender=uef6e324125cb36945f014923.

### 3.3. Cross-case data analysis and transdisciplinary approach

Over a period of one and a half year, we analysed data excerpts in a transdisciplinary team consisting of two full professors in human-centred design and autism spectrum disorders, respectively, an assistant professor in embodied interaction and a doctoral researcher focusing on co-design in the context of autism – the authors of this paper. Sessions took place on a monthly to six-week basis, guided by questions such as: How do autistic individuals experience a self-directed design process, and which needs and concerns pop up? What design decisions are the participants concerned with besides the functionality of their supportive technology?

**Figure 1.**
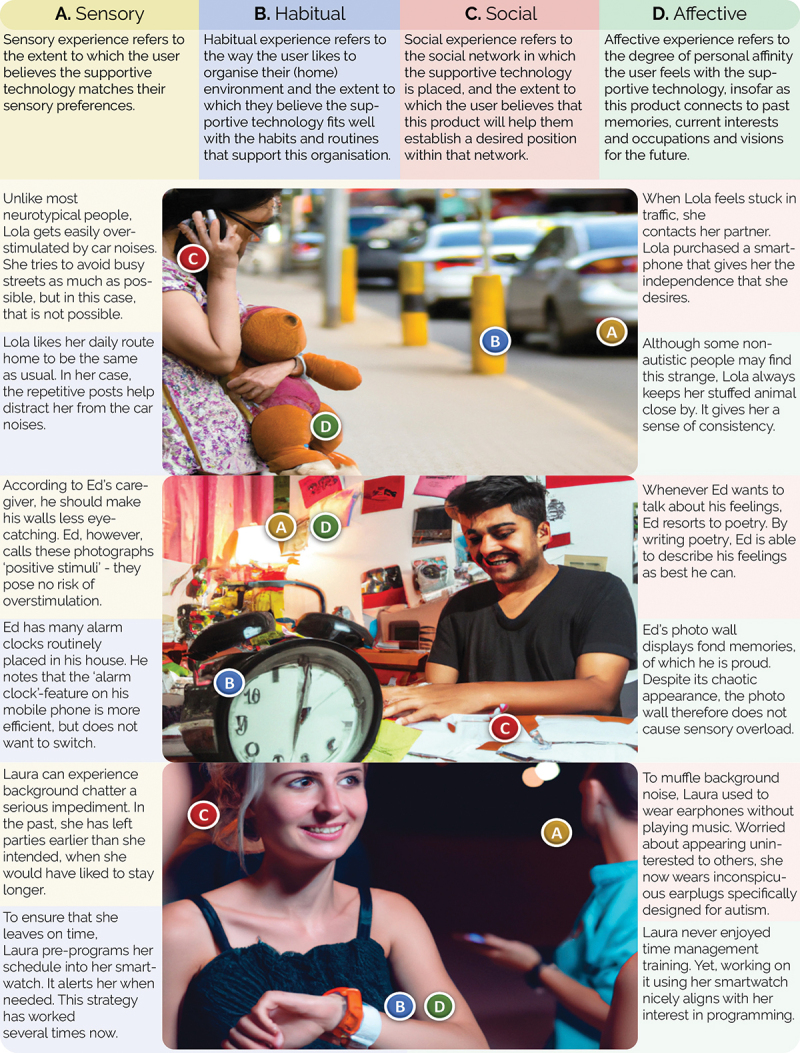
This figure shows fictional examples of how the autistic lifeworld might manifest for different people. Most of the elements are taken directly from the case studies; others are inspired by them. These images were created using DALL-E-2 (https://openai.com/dall-e-2/).

While the bottom-up input from the case studies was *steering* (Austin, Van Dijk, and Drossaert 2020), the top-down disciplinary knowledge of the scholars was used to (1) *interpret* within-case observations, (2) *identify* cross-case patterns, and gradually (3) *consolidate* the findings into the integrative framework that would become ALD. Through theory triangulation and pattern matching (Yin 2018), four dimensions of experience ‘emerged’ from the data: *sensory, habitual, social* and *affective* (Figure 1).

### 3.4. Conceptual demarcations and validity

In line with a focus on context and interaction (Section 1.2.), the habitual dimension of experience sensitised the analytical discussions and thus meant the starting point of ALD. During the case studies, however, the prominence of sensory features came to the fore, be it related to extraordinary attention to detail – e.g. Anne’s fidget spinner (Section 4.1.) – or the specific properties of an artefact that enable personal connotation – e.g. Renée’s fidget spinner (Section 4.4.). Given the ubiquity of atypical sensory experiences among autistic individuals and its relevance to design, we decided that sensory experience should be included as a separate dimension.

Following the case study with Willem (Section 4.3.), we additionally noticed that many supportive technologies are part of or inspired by formal care structures, and this plays an important role in the way supportive technology is perceived. Lastly, it was also the case study with Renée that prompted us to explore how artefacts can obtain meaning not on a practical plane, but for personal, emotional and seemingly irrational reasons. Together, these inspired us to add the social and affective dimension of experience.

We believe that these demarcations are valid, because (1) these dimensions already represent distinct research foci in autism research and design theory separately (2) yet also pertain to somewhat the same set of needs and concerns on an enactivist logic. This serves as a form of mutual verification. Nevertheless, we encourage critical reflection by scientific and autistic communities to refine and supplement ALD with another distinct dimension of experience if they believe this is necessary.

## 4. Autistic lifeworld design

In what follows, we present the results of our conceptualisation. Visual examples for each dimension are given in Figure 1.

### 4.1. Sensory experience

Atypical sensory experiences occur in as much as 90% of autistic individuals (Marco et al. 2011), and this affects the full spectrum of sensory input: static and dynamic visual stimuli tend to be more detail-oriented (Robertson and Baron-Cohen 2017), taste and olfaction are altered (Kinnaird, Stewart, and Tchanturia 2020), but also auditory and tactile perception are reportedly different from most neurotypical individuals (Espenhahn et al. 2022; Williams et al. 2021).

Although atypical sensory experiences need not be bad, for autistic persons this often concerns *hypersensitivity*. Hypersensitivity means that the individual is overwhelmed by sensory input, such as bright lights and loud sounds (Kilroy, Aziz-Zadeh, and Cermak 2019). *Hyposensitivity* is also common, which occurs when an autistic person cannot be easily energised by sensory input. Overall, sensory experiences differ greatly among autistic individuals, and it changes over time and to the extent that these experiences cause hyper- or hyposensitivity.

De Jaegher (2013) suggests an enactivist explanation, arguing that both hyper- and hyposensitivity may be coping mechanisms to deal with overstimulation, caused by difficulties with attention-shifting. Citing Gepner and Mestre (2002, 236), the world is simply ‘moving too fast’, with hypo- and hypersensitivity expressing a natural urge to slow it down – making sense of the world in a more ‘cautious’ manner, that is. For instance: pungent scents, flashing lights, loud sounds and more other stimuli require evaluative attention, yet will cause overstimulation without the right neurophysiological make-up to make sense of them.

#### 4.1.1. What does this mean for design?

**Figure 2.**
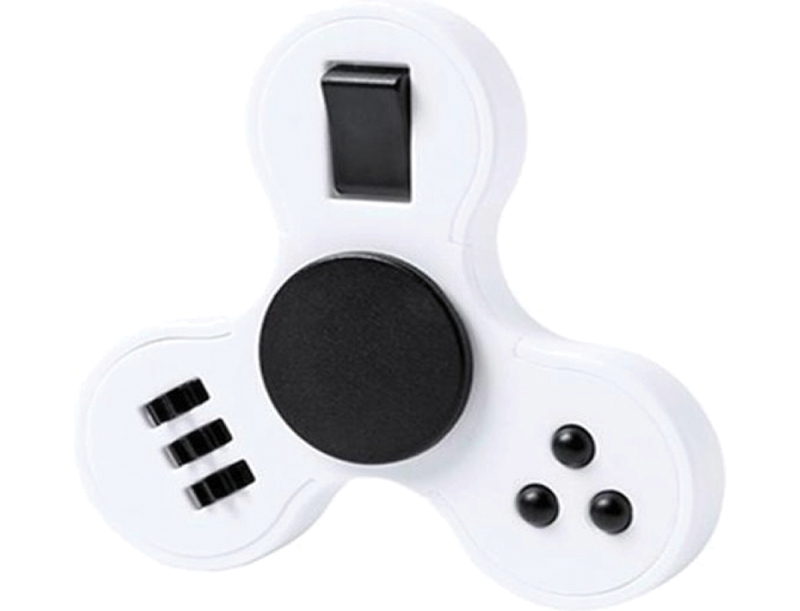
Anne was in search of the perfect fidget spinner to help her better concentrate. Among others, Anne stipulated a very specific tactile demand for her fidget spinner: different types of buttons to allow different ways of fidgeting. Facilitated by a design researcher, Anne and her caregiver conducted an internet search that led them to the fidget spinner above (source: bol.com).

Following on Lowe et al. (2014, 67), hyper- and hyposensitivity make it essential for the design of supportive technologies to provide ‘settings in which the quality of stimuli relating to sight, sound, smell and touch can be modulated to suit a person’s sensory preferences’. This became evident also in one of our case studies, given the adamance with which Anne expressed a tactile demand for her fidget spinner (Figure 2). As soon as Anne indicated that she wanted to purchase a fidget spinner, her caregiver noted that she already had a full box of fidget spinners at home. Yet Anne indicated that not just any fidget spinner would suffice to block out overstimulation; Anne used the design process to specify and look for fidget spinners that met her tactile criteria.

Attention to sensory detail is already well-recognised in the context of autism and design. Participatory methods have been developed that, for example, allow autistic people to ‘explore and test’ sensory boundaries (Gaudion et al. 2015, 54) as well as communicate their sensory preferences (Hummerstone and Parsons 2022).

Nevertheless, we observe that many user experience guidelines are still based on a clinical description of autism, expressed mostly in deficits – e.g. prioritise a ‘minimalistic aesthetic’ (Valencia, Rusu, and Botella 2021, 11) and avoid the use of bright colours (Pavlov 2014, 131). Notwithstanding the importance of these guidelines, complementary research could be done on aesthetic elements that autistic people particularly enjoy. Here, we note that the enactive approach can provide guidance. In particular, an understudied feature is the valuation of patterns, of which De Jaegher (2013, 9) notes: ‘[there] may be a value to some autistic sense-making which is simply that of enjoying or remarking on patterns – patterns in space, in ideas, in numbers, in size, in time’. In other words, autistic embodiment can enable autistic individuals to enjoy patterns in ways that non-autistic people would not necessarily understand.

### 4.2. Habitual experience

Enactivism teaches us that sense-making happens through interaction with the world, and the ontological and chronological distinction between action and perception – as separate stages in some linear information processing system – is altogether rejected. Here, an important term is that of *sensorimotor couplings*. Van Dijk writes:

The core idea is that sensorimotor couplings describe the way by which the living body continuously self-organises into coordinating patterns in response to perturbations. Coordination is established through the formation of couplings between perception (the activity of our senses) and action (the activity of our musculoskeletal, i.e., ‘motor’-system). These couplings lead to behavioural patterns (habits, routines, skills) that fit the given situation [Beer 2014]. […]. The development of a sensorimotor coupling can be seen as the development of a ‘skill’: a successful way of doing things that is stable enough to pop up when needed [Dreyfus 2002]. (2018, 11)

Within the autistic context, the idea of sensorimotor couplings relates to so-called sensory-first accounts to autism. These accounts posit that hypersensitivity is at the root of *higher-order repetitive patterns of behaviour* (Robertson and Baron-Cohen 2017). Hypersensitivity leads to altered predictive ability, the argument goes, and this could ‘motivate one to seek the most predictable situations’ (Cardon 2018, 7; Marco et al. 2011). Following an enactivist logic, autistic persons develop routines to encounter stimuli that they already know and trust, while avoiding new stimuli that require a swift shift of attention.

This resonates well with our experience with Anton, who followed a rigid ‘support system’ to memorise things with. Anton consistently used a blackboard, a calendar and small pieces of paper to write down the things he wanted to remember – often writing the same reminder on all three (Martínez Gasca 2021, 60). As he would quickly forget about these reminders as well, this support system seemed inefficient. Yet for Anton, the importance of the support system lay not in the physical notes themselves, but rather in the action of writing something down: a skilful coupling between having to remember something and writing this down to the point of memorisation. He would not deviate from it.

#### 4.2.1. What does this mean for design?

Following on Van Dijk (2018), supportive technology introduced into existing habits and routines should not go radically against them, but subtly change or add elements to them. This goes, by the way, for any physical environment and applies to any human user (Asendorpf 1980). Given the information above, such ‘fitting in’ may be essential for autistic individuals.

Participatory methods have been suggested to map said habits and routines. For instance, Pink and Leder Mackley (2012, 10) propose the use of collaborative video methods as ‘a route through which to comprehend the embodied positioning of participants in their sensory homes’. Making an explicit reference to the autistic lifeworld, Rapp et al. (2018, 56) asked autistic participants to sketch a map of the city centres and neighbourhoods in which they lived – not to obtain an accurate description of said spaces, but rather to understand how they perceived the ‘important landmarks’ and ‘routes they habitually travelled’.

**Figure 3.**
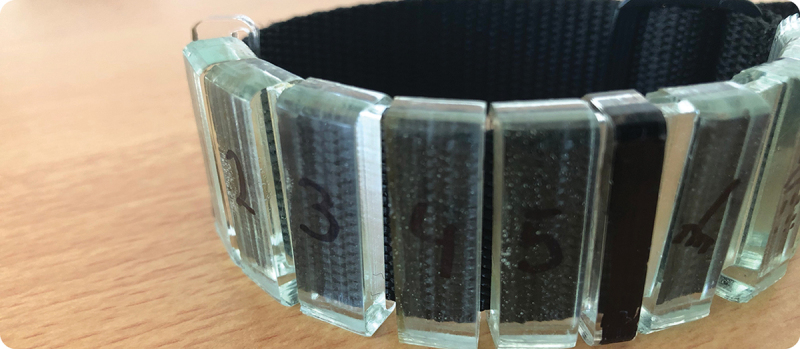
Anton ended up creating a bracelet that would be an addition to his ‘support system’, not a replacement (Martínez Gasca 2021, 73). The bracelet offered a physical reminder and pushed him to prioritise the reminders he wanted to attend to.

Anton went on to design a ‘reminder bracelet’ that complemented his support system, not replace it (Figure 3). Instead of going radically against his own routines, Anton figured that he could best alter them by introducing new sensorimotor couplings and gradually changing his habits along the way.

### 4.3. Social experience

In this section, we extend the enactivist notion of autonomy to the social realm. Earlier, we referred to ‘needful freedom’ (Loaiza 2019): individuals will assess their interactions with others in terms of how much they contribute to their autonomy.

In the context of autism, supportive technologies mediate social relations between autistic people and their caregivers in a care context, but also embody societal beliefs on what autism is and how it should be treated accordingly. Especially illustrative are those technologies that build on Applied Behaviour Analysis (ABA) (e.g. Artoni et al. 2018; Chow 2021), of which some critics say frames autism predominantly as a medical condition that needs to be mitigated, rectified or even ‘cured’ (Kirkham 2017). In line with ABA, these supportive technologies aim to support ‘normal’ behaviour – such as looking another in the eyes while speaking (Palestra et al. 2016) – and discourage ‘abnormal’ behaviour – such as hand flapping or other motor stereotypies (Malhotra et al. 2010). Others have criticised autism’s stereotypisation, stigmatisation (Treweek et al. 2019) and infantilisation (Stevenson, Harp, and Gernsbacher 2011). Spiel, Frauenberger and Fitzpatrick write:

The implicit assumption is that there is an increase in quality of life for autistic individuals if they function in a more neurotypical way and technologies are designed towards this goal. Hence, most of these technologies are embedded into attributes that a neurotypical society deems important and meaningful. (2017, 52)

**Figure 4.**
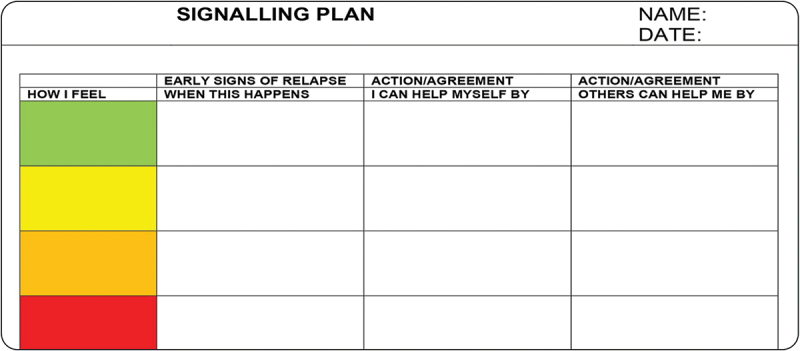
The signalling plan is a reflection tool initially developed to prevent aggression in psychiatric hospital wards (Van der Werf and Goedhart 1998), and since then has been adapted to the autistic context as well. Using a colour code (green, yellow, orange or red), the user is asked to identify and indicate early signs of relapse to prevent moments of crisis (translated from Dutch).

Although more subtly and in different ways, critiques of stereotypisation, stigmatisation and infantilisation also emerged during the design case studies. For example, Willem expressed dissatisfaction with the ‘signalling plan’ because it would automatically link any frustration he experienced to his autism, when there could have been a legitimate reason behind it (Figure 4). He also disliked the ‘labelling’ of autism that he felt was embedded in the signalling plan. Much to Willem’s dismay, the signalling plan had been a mandatory part of his autism coaching.

#### 4.3.1. What does this mean for design?

Given the above, designers should observe that supportive technologies are not introduced in a social vacuum. Rather, they are embedded in and may change social, cultural and political ties between caregivers, autistic individuals, and ultimately society as a whole. This makes it that designers should be well-attuned to the social network in which their supportive technology is going to be inserted, e.g. through stakeholder mapping and analysis (Knowles and Spencer 2016) and multi-stakeholder engagement (Andersen and Mosleh 2021).

In addition, supportive technologies designed specifically for communicative purposes should respect different ways of and preferences for communication (e.g. Hummerstone and Parsons 2022). Willem, for example, favoured the use of poetry. Text, he argued, was best able to capture his feelings. Alternatively, Klein (2022) suggests that photography as a form of visual communication can help autistic individuals share more effectively how they experience every-day life.

### 4.4. Affective experience

**Figure 5.**
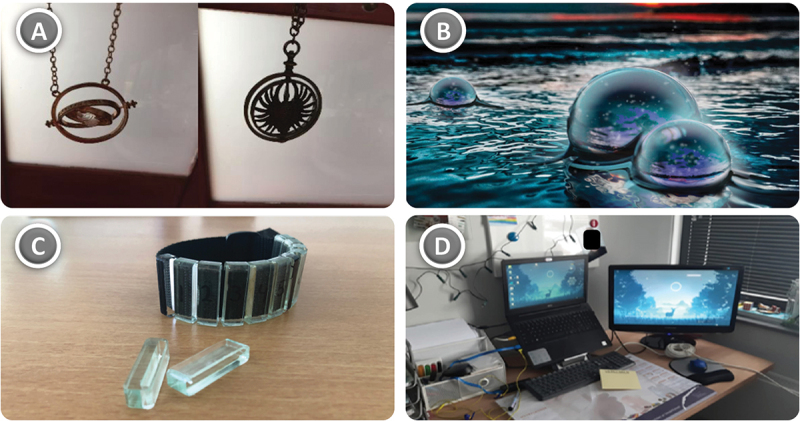
(A) Two fidget tools owned by Renée, the right one being referred to in the text. Renée claimed that the left one was better for fidgeting due to its two axes, but she nonetheless preferred the right one due to the emotional value behind it. (B) A water crystal designed by Overdevest (2021) for case study participant Simon’s fictional world ‘Aether’. (C) Anton’s prototype for a bracelet supporting self-management. (D) Willem’s work setting at home, including the laptop referred to in the text.

Sense-making pertains to one more level of evaluative interaction: emotion and affect. During the case studies, it was observed that many participants preferred to develop and use supportive technologies to which they were emotionally connected, in one way or another. For instance, Renée explained that, of the two fidget tools that she used for suppressing sensory overstimulation, her favourite was one that she had purchased at an amusement park that she had visited with her friends (Figure 5(A)) (Wonink 2021).

Similar stories exist for the other participants: technologies that not only connect to memories from the past, but also current interests and visions for the future. On behalf of Simon, for example, Overdevest (2021) used 3D creation tool Unreal Engine to create a fictional world called ‘Aether’ (Figure 5(B)). Simon’s design challenge was to gain more confidence and, together with his caregiver, Simon decided that his special interest in high-fantasy could be used motivationally: as a way to showcase his unique skills and perseverance. Likewise, Anton’s prototype for a self-management bracelet matched his tinkering skills with plexiglass (Figure 5(C)). More profoundly, Willem identified his laptop as ‘an extension of himself’ (Figure 5(D)). As he has always been rather slim, the study books in high school were often too heavy to be carried around. In this respect, the laptop – and a NeoPad before that – enabled him to nonetheless participate as any other student. In response, the design researcher suggested finding a digital solution, after which they experimented with a management system that matched Willem’s interest in programming. Currently, Willem is preparing for a career in ICT.

Special interests and occupations are enjoyed by 75 to 95% of autistic individuals (Grove et al. 2018), positively associated with self-stimulation and personal validation, and described as possible coping strategies for autistic individuals to reduce anxiety (De Jaegher 2013). In addition, emotional couplings to objects of use are believed to be even stronger in comparison to non-autistic individuals. Zamoscik et al. (2016, 6) write: ‘[since] people with autism show heightened responses to sensory stimulation […], memory processing via sensory features might be enhanced in adults with autism, which would facilitate retrieval of those memories using sensory pathways’. Overall, this could mean that the autistic lifeworld does not only consists of *more* evocative objects than the lifeworld of a non-autistic individual, but that these objects are – in line with Heersmink’s work on ‘distributed selves’ (2017, 2018) – also *stronger* scaffolds to emotionally meaningful aspects of someone’s life and identity.

#### 4.4.1. What does this mean for design?

Designers should pay attention to the personal significance that every-day objects and their features can have in the lifeworld of an autistic individual, and be aware that these can be effectively used in a collaborative design process. A good example of this is the aforementioned *OutsideTheBox*-project by Frauenberger, Spiel, and Makhaeva (2019), in which the design researchers used participatory methods to identify and centralise the special interests of their participants in the design process. This resulted in, for instance, an alarm clock shaped like a character from SuperMario, and a reflection device in the style of scientific instruments.

Yet, it is also here where co-design seems to reach its methodological borders. While current interests and occupations are relatively easy to identify, we found that the ‘emotional landscape’ of our participants remained largely inaccessible. For instance, when Anne looked at several fidget spinners that met her tactile criteria, she did not seem to immediately understand herself *why* she liked some more than others, although it was clear she was assessing them against a background of previous experiences with other fidget tools. Taking this a step further, we felt lucky that Renée had gotten aware of her amusement park fidget spinner, claiming that the ‘[co-design] assignment had made her really consider the items around her in ways she had never before’ (Wonink 2021, 69). It would have made sense if the fidget spinner had escaped her attention altogether: the fidget spinner already reached seamless and purposeful integration into her lifeworld, especially because it was perceived so positively.

We will briefly return to this in Section 5.2., as this observation is also relevant with regard to the other dimensions of experience. We now turn to the wrap-up of this paper, beginning with reflections on our methodological approach.

## 5. Methodological reflections and future research

### 5.1. Methodological reflections

One limitation of our approach is the relative *homogeneity* of our research participants. We note that we have only worked with autistic young adults for whom (semi-) independent living is a viable goal, and the frame and examples of supportive technologies provided in this paper also fit that pool of participants. For example, none of our participants were dealing with strongly confined motor skills, severe difficulties with communication or – as stated in Section 3.1. – co-occurring intellectual disability. Autistic individuals with high support needs are not only underrepresented in co-design, but also more generally in the discourse on neurodiversity (Den Houting 2019). On reflection, we expect that our framework does not represent all subgroups of the autistic spectrum, and further research is needed to determine how it can be used by autistic individuals from different age groups and with different levels of comorbidity.

This brings us to the second methodological limitation of our study: the absence of *clinical* research. To expand the reach of ALD, it needs to find stronger integration in the healthcare context and be complemented by clinical research and already existing frameworks for therapy and (behaviour) intervention. In line with neurodiversity, these frameworks should be participatory and autism-led (Pukki et al. 2022), focusing on capabilities at the side of the individual (Pellicano et al. 2022) and improving compatibility at the side of their environment (Doyle 2020). Traditional approaches may also be used, as shown by Wilson et al. (2019) who use Speech-Language Therapy to involve minimally-verbal autistic children in co-design. Clinical research can help assign a greater role to professional caregivers as design partners, to assist autistic participants in making sense of the design activities; generate and express design ideas.

Finally, we believe that autistic individuals could have been more thoroughly involved in the *analytical* phase of this study, instead of only at the start and during the research (Section 3.2.). In retrospect, including an autistic stakeholder in the analytical team would have allowed neurodiverse interpretations of the data (Fletcher-Watson et al. 2019), validating the extent to which ALD resonates with their own lived reality and perhaps that of other autistic peers.

### 5.2. Directions for future research

Central to ALD is the enactive approach, recalling De Jaegher’s description of it as a ‘dialogue between phenomenology and science’ (2013, 6). With respect to our framework, directions for future research can be suggested on both sides.

We begin by highlighting the importance of participatory methods in (1) enabling designers to identify valuable information about their participants’ needs and concerns and (2) enabling participants to share this information in creative ways – i.e. poetry and photography (Section 4.2.). Equal attention should be given to tools and techniques that enable autistic participants to better understand their *own* lifeworld, which we discovered is often not the case. As seen in the previously mentioned *MyDayLight*-project (Van Dijk and Hummels 2017; Van Dijk et al. 2019), such self-understanding can be achieved through reflection prompts and iterative design cycles, in which participants learn more about themselves as they go through a design process and reciprocally adapt the technology based on insights gained along the way. Following on Section 4.4., the challenge is to make participants aware of habits, daily-life strategies and devices that are already successfully in use; that have ‘faded into the background’ but could otherwise have meant the starting point of a design process. Which tools and techniques can be devised to bring out precisely these invisible elements?

On the other side of the dialogue, autism research can continue to address questions that ALD gives rise to but have remained unanswered in this paper. For example (Section 4.1.): How should we understand the appreciation of patterns by autistic individuals, and could this inform an expansion of the participatory design space to include other aesthetic elements that may be valued for the same reason, such as visual resemblance and attention to detail? Or (Section 4.4.): why do autistic people tend to anthropomorphise every-day objects (White and Remington 2019), and what is the (normative) relevance of this for supportive technology design? For instance, would it not be an effective strategy to give supportive technology human-like features? But what happens if the technology breaks down or is stolen?

Enactivism finds itself in the middle, serving as an underlying research philosophy but also as an ethical safeguard. By linking to phenomenology, enactivism helps ensure that the value of autism research – whether studied as a disability or biological difference – is always considered insofar as it contributes to better understanding, recognition and empowerment of autistic individuals in real-world contexts. For instance, as shown in the research presented, to inform the development of personalised supportive technology. This, again, highlights the importance of participatory research.

## 6. Conclusion

This paper started with the observation that supportive technology design has a strong focus on limitations assumed in the deficit model of autism. There has been a growing call for designers to instead get a better handle on how to integrate the full richness of the autistic lifeworld into the design space.

To this end, we presented *Autistic Lifeworld Design*. Through four dimensions of experience, ALD provides designers with a hands-on overview of how the autistic lifeworld can be understood and how to account for it in a participatory design process. Here, concepts taken from enactivism – e.g. *sense-making, evaluative interaction* and *embodiment*, among others – enabled us to reframe the way in which technology can be supportive, namely by helping to sustain different levels of homoeostasis.

To conclude, ALD offers a novel lens that allows designers to put the lived experiences of autistic individuals at the centre of the design process, with special attention to the role of bodily structures and processing in shaping these experiences – a valuable source of autism-specific scientific research that we believe has so far not been fully exploited in existing co-design research.

## Notes

1. Following on Baron-Cohen (2017, 745), disability requires ‘societal support, acceptance of difference and diversity, and societal “reasonable adjustment”, while disorder is usually taken to require cure or treatment’. Since neurological biomarkers are not reliably available, diagnosis of autism spectrum disorder largely relies on behavioural tools that are influenced by cultural expectations of normal human behaviour (Dyck and Russell 2020). This causes that an autistic person only warrants a diagnosis if they experience disability in every-day life as a result of their autism.

2. Participants and caregivers were informed of this criterion before participation, which was established to avoid confusing autism and intellectual disability in the context of our framework. However, we are aware of the high prevalence of co-occurring intellectual disability among autistic individuals (33%) as well as the different ways in which autism and intellectual disability might overlap diagnostically (Werkman et al. 2022).
